# Validity, reliability and precision of a novel virtual reality rod and disk test to assess visual dependence

**DOI:** 10.1038/s41598-026-45536-8

**Published:** 2026-03-23

**Authors:** Yiduo Wang, Caroline M. Alexander, Paul H. Strutton

**Affiliations:** The Nick Davey Laboratory, Division of Surgery, Department of Surgery and Cancer, Faculty of Medicine, Sir Michael Uren Hub, Imperial College London, White City Campus, 86 Wood Lane, London, W12 0BZ UK

**Keywords:** Visual Dependence, Postural Control, Virtual Reality, Balance Disorders, Perception, Proprioception, Diseases, Health care, Medical research, Neurology

## Abstract

Visual dependence (VD) is heightened in pathologies where balance is compromised. It is measured using the Rod and Disk Test (RDT), traditionally delivered through a personal computer (PC). This restricts settings under which the test can be taken. This study aimed to evaluate validity, reliability and measurement precision of a novel virtual reality (VR) RDT. VD of 15 healthy participants and 15 people with symptomatic hypermobility was assessed using VR- and PC-based RDT to evaluate convergent validity. Assessments were conducted in three head positions. Intra-rater reliability was assessed by measuring VD of 15 healthy participants seven days apart. Convergent validity was evaluated using correlation analysis; intra-rater reliability using intraclass correlation coefficients. Bland-Altman plots assessed agreement, and measurement precision was reported as standard error of measurement (SEM) and minimal detectable change (MDC). Weak to moderate convergent validity was seen between VR- and PC-based tests, with coefficients of 0.3, 0.4 and 0.3. PC-based RDT showed poor to moderate ICCs; the VR-based RDT demonstrated poor ICCs. SEM and MDC for PC ranged from 0.44–0.47° and 1.23–1.30° respectively; for VR they ranged from 0.65–0.78° and 1.81–2.17° respectively. VR-based RDT demonstrated weak to moderate convergent validity and poor intra-rater reliability.

**Supplementary Information:**

The online version contains supplementary material available at 10.1038/s41598-026-45536-8.

## Introduction

Postural control, defined as the ability to maintain balance and spatial orientation, requires the integration of visual, vestibular, and proprioceptive inputs^1,2^. How multisensory systems contribute to postural stability in different clinical populations has been extensively investigated, particularly in conditions where vestibular or proprioceptive control are compromised or absent^3–5^. In this context, visual input compensates when other sensory inputs are unreliable^6^. The degree to which visual cues are reweighted to compensate for altered proprioceptive or vestibular input is termed visual dependence^7^.

People with vestibular disorders typically exhibit increased visual dependence, which can lead to visual vertigo, manifesting as imbalance in response to visual motion^8^. Increased visual dependence has also been associated with aging^6^, dizziness^9,10^, anxiety^11,12^, motion sickness^13,14^, migraine^15^, and is linked to an increased risk of falls^16^. Quantifying visual dependence in people with balance disorders is therefore important to understand some of the underpinning mechanisms and may facilitate targeted rehabilitation strategies, such as exposure to optokinetic stimulation to reduce visual dependence^17,18^.

Since its introduction in 1948^19^, the umbrella term, *Rod and Disk Tes*t (RDT), has been widely used to assess visual dependence and related concepts^9,20,21^. Past clinical studies performed assessments by requiring participants to look at and adjust a movable bar to indicate when it was perceived to be vertical, while different visual contexts are presented; these included a static tilted frame, a rotating disc, the use of a bucket or hemispheric dome screens^22,23^. Subsequently, the test was adapted so that it could be delivered through a PC to enhance standardization^9,24,25^. More recently, virtual reality (VR)-based versions of the RDT have been developed^26–28^. VR would enable easier assessment of visual dependence in varied head positions to manipulate both vestibular^27,29,30^ and proprioceptive input. It would also enhance healthcare by enabling bedside or remote assessments or follow-up tools through improved portability, and potentially supporting the broader transition towards digital health^31–33^. Despite claimed clinical generalisability, previous studies have not rigorously validated the VR-based RDT^26–28^ against an established reference test, i.e. the PC-based RDT. Furthermore, these studies have not assessed validity, reliability and precision together. This limits VR-based RDT adoption in both clinical and research settings. Without these metrics, clinicians cannot determine if a patient’s change in score is due to recovery or measurement error.

While the role of visual dependence has been well studied in vestibular and other neurological diseases^8–10,15^, less attention has been given to other populations with impaired balance^34^. One such population that we are interested in, is people with symptomatic hypermobility^35^. This is characterised by a wide range of multisystemic symptoms^36^, including poor balance and falls^35,37–39^. To what extent visual dependence contributes to imbalance remains unexplored in this cohort^35^.

To address these gaps, the purpose of this study was to assess a convergent validity of an in-house designed VR-based RDT against the accepted PC delivered version. The purpose was also to understand intra-rater test-retest reliability and measurement precision of both methods, to support future research exploring their clinical and research utility.

## Methods

### Study design

A laboratory-based validation and test-retest design was employed. Data collection took place from December 2023 to December 2024. The study comprised three components: (i) a convergent validation assessment, (ii) test-retest reliability assessment, and (iii) evaluation of measurement precision for both PC- and VR-based versions. This study was conducted in accordance with the principles of the Declaration of Helsinki and approved by Imperial College Research Ethics Committee (Reference No. IC6700016). All participants provided written, informed consent prior to participation. Data were anonymised and securely stored.

## Participants

An a priori power analysis was performed in G*Power (version 3.1.9.7) using the bivariate correlation model^40^. For convergent validation, assuming ρ = 0.50, α = 0.05 (two-tailed), and power = 0.80 in line with a previous validation study^9^, the required sample was 29 participants in total. For intra-rater reliability, assuming ρ = 0.60 with the same α and power in line with a previous reliability study^41^, the required sample was 15 participants. To reduce the risk of missing data, a total of 30 participants was recruited. Healthy participants were conveniently recruited via social media and university research portals. Participants with symptomatic hypermobility were purposively recruited through specialised organizations (e.g., Ehlers Danlos Support UK, Hypermobility Syndromes Association).

Inclusion criteria were: (i) 15 healthy adults between 18 and 50 years of age, with normal or corrected-to-normal vision and no reported screen-related discomfort or motion sickness; (ii) 15 adults between 18 and 50 years of age, with Hypermobility Spectrum Disorder (HSD) or hypermobile Ehlers Danlos Syndrome (hEDS). They were classified according to the 2017 international classification^42^ which includes the Beighton score (≥ 5/9)^43^; the Brighton criteria (either 2 major, 1 major and 2 minor, or 4 minor)^44^ were also applied.

Exclusion criteria were: (i) current diagnosis of other musculoskeletal or neurological disorders; (ii) known vestibular or auditory impairments; (iii) any genetically confirmed connective tissue disorders (e.g. Marfan syndrome, osteogenesis imperfecta, Down syndrome, Loeys-Dietz syndrome and other subtypes of Ehlers-Danlos Syndrome); (iv) any medical condition unrelated to HSD or hEDS.

## Experimental procedures

Visual dependence was assessed in all participants using both the PC-based RDT in line with previous studies^9,45^ and the VR-based version in random order to assess validity. Cybersickness in VR was evaluated using a questionnaire following the completion of the VR-based RDT. Intra-rater reliability across both PC-based and VR-based systems was assessed by measuring visual dependence of 15 healthy participants approximately seven days apart in the same way.

### PC-based RDT

**Fig. 1:**
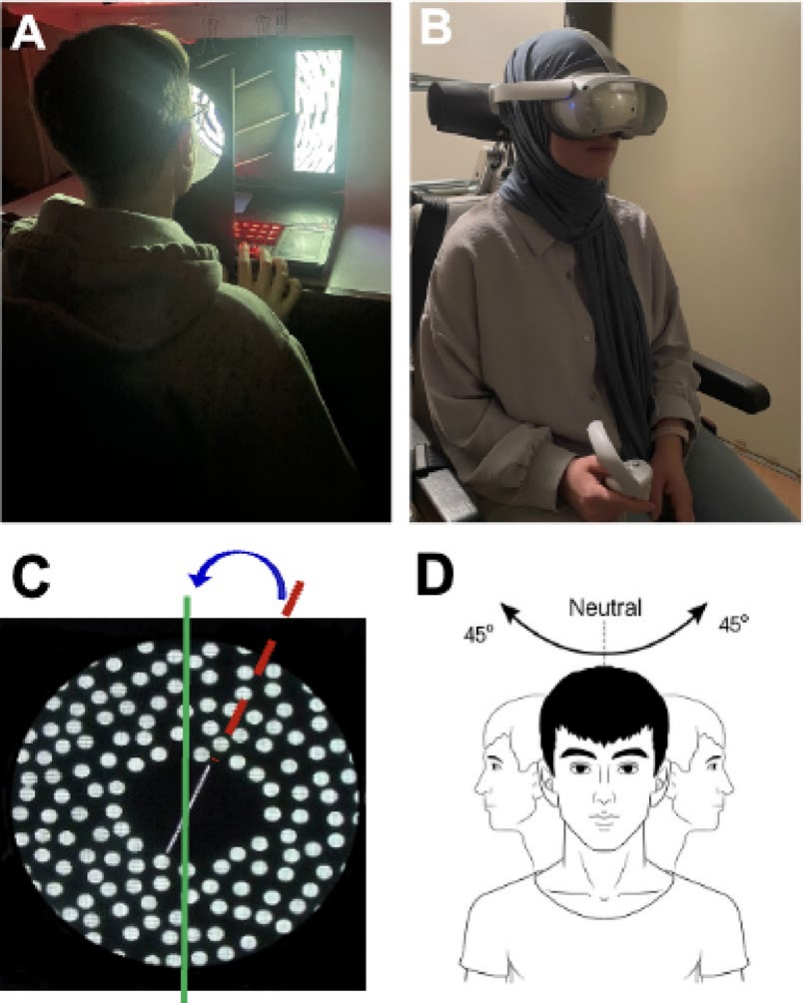
Setup of the Rod and Disk Test for visual dependence. **A**. The set up for the PC-based Rod and Disk Test. **B**. The set up for the VR-based Rod and Disk Test. **C**. Subjective visual vertical error, defined as the angular deviation between the true vertical and the participant’s positioning of the rod. **D**. Participants repeated the test with head rotated 45° left and 45° right in the yawn plane. All participant photographs are reproduced with consent. Illustration D was produced by authors using Adobe Photoshop for the purpose of this study.

Participants were seated in a darkened room and viewed a PC screen through a 30 cm deep cone at the eyes (15 cm diameter aperture; 39° visual field; see Fig. 1 A). Each trial displayed a white rod (6 cm long) on a black background, capable of being rotated 360° around its midpoint. Surrounding the rod, 220 off-white dots (8 mm in diameter; 1.5° visual field) were randomly distributed to create static or dynamic visual scenes. The rod was randomly placed at an angle of ± 20°, ± 40°, or ± 70° from vertical, with timing controlled by the researcher^9^. Participants then used a keyboard to align the rod to their perceived vertical (see Fig. 1 C) under three visual conditions: (i) static dots, (ii) dots rotating at 30°/s clockwise, (iii) dots rotating at 30°/s anticlockwise; the dynamic conditions were presented in random order after the static condition. Each condition comprised three to five trials, with a total of 12 trials. Testing was repeated with the head in three positions: neutral, 45° left rotation, and 45° right rotation in the yaw plane (see Fig. 1D). Head angles were measured using a goniometer. The PC-based RDT software is publicly available at: https://www.imperial.ac.uk/brain-sciences/research/neurology/vestibular-neurology/.

### VR- based RDT

The VR-based test was implemented on a Pico 4 All-in-One headset (Pico Immersive, Beijing, China). Prior to testing, participants were instructed to sit in a relaxed, neutral and upright position, holding a controller in their dominant hand (see Fig. 1B). To ensure precise alignment within the three-dimensional environment, participants pressed and held the ‘Home’ button on the controller for 2–3 s. This function recalibrated the environment, aligning the test graphics with the participant’s trunk, and adjusted the display to replicate the visual distance of the PC-based version. A white rod then appeared at the centre of the visual field to assist participants in aligning their head and eyes accurately before each condition. This cue was intended both to standardise head position and to capture three-dimensional postural variation prior to testing.

Rod adjustment was performed using the controller: the ‘X’ or ‘A’ button to initiate or confirm alignment, and the thumb stick to rotate the rod. Visual conditions matched the PC-based test. Participants were instructed to align the rod to their perceived vertical under three conditions: static dots, dots rotating at 30°/s clockwise, and dots rotating at 30°/s anticlockwise. Each condition consisted of four trials, and the two dynamic conditions were presented in random order following the static condition, yielding a total of 12 trials. At the beginning of each trial, the rod was positioned randomly at ± 20°, ± 40°, or ± 70° from vertical to match the PC-based version. All procedures were supervised by a trained researcher. The application was terminated by pressing the ‘Home’ button and selecting ‘Exit’ from the system menu.

### Cybersickness in VR questionnaire

The questionnaire was filled out at completion of the VR-based test. The Cybersickness in VR questionnaire (CSQ-VR) consists of six items, each rated on a seven-point Likert scale, designed to measure three core symptom domains: nausea, vestibular and oculomotor symptoms^46^. Each domain is evaluated through two items. Sub-scores are calculated for each domain, and these are combined to produce an overall score from a minimum value of 6 to a maximum value of 42, which represents cybersickness severity in VR.

### Data analysis

Data were extracted using Spyder Python (version 5) and analysed and visualised using GraphPad Prism 10.0 (GraphPad Software Inc., USA). Data normality was assessed using the Shapiro-Wilk test. Cybersickness scores were summarised using mean and standard deviation (SD). A significance level of α = 0.05 was adopted, and medians with 95% confidence intervals are reported where appropriate.

### Visual dependence

Subjective visual vertical error ($\:{SVV}_{error\:}$) was calculated for each trial as the angle of the rod aligned by the participant with respect to vertical (0° - gravitational vertical). The mean SVV error across $\:n$ trials in each condition ($\:{\stackrel{-}{SVV}}_{condition}$) was calculated using formula I, where $\:n=4\:$trials in VR-based version and $\:n=3\sim5$ trials in PC-based version. A complete RDT consisted of a total of 12 trials including three conditions (i.e. static dots, dots rotating clockwise and dots rotating anticlockwise). Visual dependence was calculated as the difference between the mean of the dynamic SVV conditions (dots rotating clockwise and anticlockwise) minus the static SVV condition (static dots) using formula II, in line with previous research^47^.

I $${\overline {SVV} _{condition}} = \;\frac{1}{n}\;\mathop \sum \limits_{i = 1}^n \left| {SV{V_{error}}} \right|$$

II $$Visual\;Dependence\; = \;\frac{{{{\overline {SVV} }_{clockwise}} + \;{{\overline {SVV} }_{anticlockwise}}}}{2} - \;{\overline {SVV} _{static}}$$

### Validation analysis

Convergent validity was assessed by correlating visual dependence derived from the VR-based and PC-based RDTs. Correlation analysis (Pearson’s correlation coefficient for normally distributed data, or Spearman’s Rank coefficient non normally distributed data) was used to quantify the association between visual dependence scores obtained from the two systems, thereby examining the extent to which the VR-based RDT reflects the same construct as the established PC-based method. Bland-Altman analysis was performed to examine mean bias and limits of agreement between the two systems^48^. Correlation strength (r/rho) was classified as: perfect (± 1), strong (± 0.7–0.9), moderate (± 0.4–0.6), weak (± 0.1–0.3), or none (0)^49^.

### Reliability analysis

Intra-rater test-retest reliability of both PC-based and VR-based RDTs was evaluated using the intraclass correlation coefficient (ICC), derived from visual dependence measured at two sessions separated by at least one week. ICCs were calculated using a two-way mixed-effects model for single measurements [ICC^1,3^^50^. The strength of ICCs was categorised as: poor (< 0.50), moderate (0.50–0.75), good (0.75–0.90), or excellent (> 0.90)^50^. The standard error of measurement (SEM) and minimal detectable change (MDC) were also calculated for both systems using formula III and IV, respectively^51,52^.

III $$SEM = SD\sqrt {1 - ICC}$$

IV $$MDC = 1.96*SEM*\sqrt 2$$

## Results

Thirty participants were included in the study (mean age = 28.7 years, SD = 7.4); the sample was predominantly female (80%) and right-handed (90%). Fifteen participants demonstrated symptomatic joint hypermobility. Demographics of thirty participants are presented in Table 1.

**Table 1:** Participant characteristics.

Category	Details
Age (years, mean ± SD)	28.7 ± 7.4
Sex	Female: 24 (80%); Male: 6 (20%)
Handedness	Right: 27 (90%); Left: 2 (6.7%); Mixed: 1 (3.3%)
Beighton score (mean ± SD)	Hypermobile participants: All ≥ 5/9; 6.4 ± 1.24
2017 hEDS International Classification	HSD: 13 (87%); hEDS: 2 (13%)
Brighton criteria	2 major: 12 (80%); 1 major and 2 minor: 3 (20%)

Abbreviations: HSD - Hypermobility Spectrum Disorder; hEDS - hypermobile Ehlers Danlos Syndrome.

### Validation

**Fig. 2:**
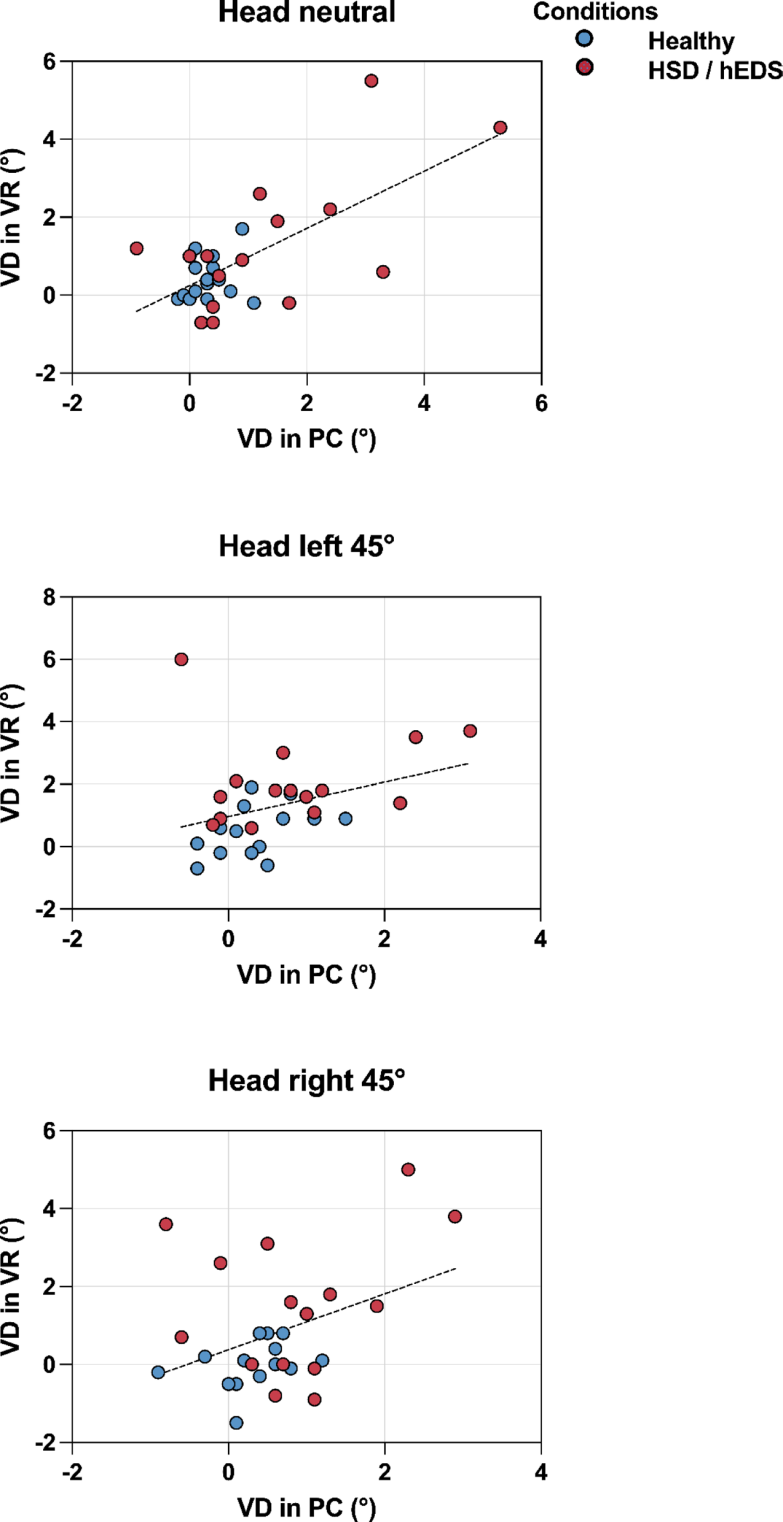
Visual dependence measured with PC- and VR-based Rod and Disk Tests Visual dependence delivered using the PC-based and VR-based tests in three head positions; neutral, head rotated 45° to the left and head rotated 45° to the right. Linear regression lines are shown; individual data points show visual dependence pairs: blue represents healthy participants; red represents people who are hypermobile.

The strength of association between visual dependence derived from the PC-based and VR-based RDTs varied across head positions. The data were not normally distributed. Spearman’s correlation coefficients were *r* = 0.3 for the head-neutral position (*p* = 0.06), *r* = 0.4 for head rotated 45° to the left (*p* < 0.05), and *r* = 0.3 for head rotated 45° to the right (*p* = 0.16). These results indicated a significant moderate correlation when the head was rotated 45° to the left, whereas correlations were not significant whilst the head was neutral and was rotated to the right (Fig. 2).

**Fig. 3:**
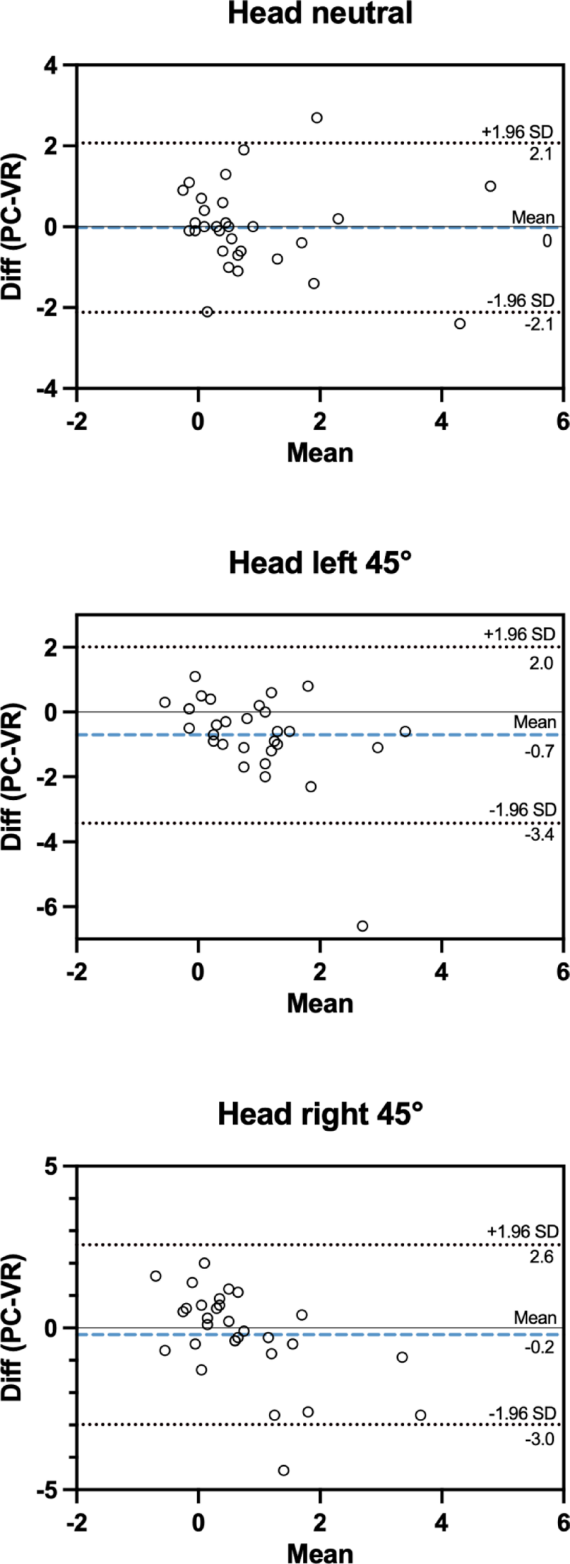
Bland-Altman plots for all head positions. Bland-Altman plots show PC-VR measurement differences under the three head positions. Diff - Difference; SD - Standard deviation; PC - Personal Computer; VR - Virtual Reality. For the head-neutral position (top), the mean difference was 0° and 95% limits of agreement (LoA) were [–2.1°, 2.1°]. For the head rotated 45° to the left (middle), the mean difference was − 0.7° (LoA [–3.4°, 2.0°]), and for the head rotated 45° to the right (bottom), it was − 0.2° (LoA [–3.0°, 2.6°]). Dashed lines (blue) show the mean and dotted lines (black) show limits of agreement.

To further assess the level of agreement between measurement systems, Bland-Altman analyses were performed. The mean difference (PC-VR) was 0°, − 0.7°, and − 0.2° for the head-neutral, left-rotated, and right-rotated conditions respectively. These findings suggest an overall trivial overestimation of visual dependence by the VR-based system; however, it should be noted that the overestimation is less than 1°. The 95% limits of agreement were: head neutral: −1.9° to 1.7°; head rotated to the left: −3.0° to 1.6°; head rotated to the right: −2.5° to 1.8° (Fig. 3).

### Reliability

Test-retest reliability of the PC-based and VR-based RDT was evaluated across the three head positions in healthy adults. The VR-based system showed poor intra-rater test-retest reliability in neutral, left and right head positions (ICC = 0.02, 0.16, 0.04 respectively). The PC-based RDT showed poor to moderate intra-rater test-retest reliability in neutral, left and right head positions (ICCs = 0.26, 0.35, 0.47 respectively). However, it is interesting to note that the MDCs were small; approximately 2°. ICC, MDC and SEM details are presented in Table 2.

**Table 2:** Test-retest reliability and precision of PC and VR-based systems.

Variables	VR-based RDT				PC-based RDT			
	SD	ICC	SEM	MDC	SD	ICC	SEM	MDC
		(95%CI)				(95%CI)		
VD (°) (Head Neutral)	0.78	0.02	0.76	2.11	0.55	0.26	0.47	1.30
		(−0.48–0.52)				(−0.27–0.67)		
VD (°) (Head Left 45°)	0.88	0.16	0.78	2.17	0.58	0.35	0.44	1.23
		(−0.36 −0.61)				(−0.17- 0.72)		
VD (°) (Head Right 45°)	0.66	0.04	0.65	1.81	0.66	0.47**	0.47	1.29
		(−0.46–0.53)				(−0.03–0.78)		

Abbreviations: VD – visual dependence; VR – virtual reality; RDT – Rod and Disk Test; PC – personal computer; ICC – Intraclass Correlation Coefficient; CI – Confidence Interval; SEM – standard error of measurement; MDC – minimal detectable change. ** *p* < 0.05.

Cybersickness questionnaire in virtual reality.

Cybersickness levels for all participants were low (mean ± standard deviation; 13.6 ± 7.64). Sub-scores of the questionnaire for each group are provided in Table 3.

**Table 3:** Cybersickness questionnaire in VR scores.

Category	Participants (*n* = 30)	Healthy (*n* = 15)	Hypermobile (*n* = 15)
Nausea	4.0 ± 2.44	2.7 ± 0.90	5.4 ± 2.75
Vestibular	4.0 ± 2.63	2.7 ± 0.82	5.3 ± 3.13
Oculomotor	5.5 ± 3.60	3.3 ± 1.23	7.7 ± 3.86
Total Score	13.6 ± 7.64	8.7 ± 1.72	18.5 ± 8.15

All data are presented as mean ± standard deviation.

## Discussion

This study evaluated convergent validity of the VR-based RDT against the frequently used PC-based test for measuring visual dependence. Our findings demonstrate weak to moderate correlations across all head positions, with only a moderate correlation when the head was rotated to the left. Intra-rater test-retest reliability was poor across all head positions in the VR-based RDT and poor to moderate in neutral, left and right head positions in the PC-based RDT. Measures of precision were also explored and parameters revealed that the precision of the VR-based test was approximately 2°; that is, there needs to be a change that exceeds approximately 2° to be considered real. All participants (even in people with hypermobility) reported low cybersickness during the task undertaken in VR. Consequently, cybersickness was not analysed as a covariate attribute to lack of statistical variance.

### Validity

Two factors likely contributed to the finding of weak to moderate correlations between our novel VR-based RDT and the established PC-version. On the one hand, to improve comparability, we changed the usual resolution of the PC from 2° to 0.1° to match the resolution of the VR-based system, which may have amplified minor differences; this might explain, in part, the low correlations. In addition, despite including participants we hypothesised would have high visual dependence (people with symptomatic hypermobility), the range of visual dependence was smaller than we anticipated (PC: 0.9° − 5.3°; VR: 0.7° − 5.5°), compared to people with vestibular disorders^9^. With such a limited range in visual dependence, the low correlation derived is due to it being based upon small degrees of error in the two systems. It is important to note that the reliability of the tests could impact the tests of validity^53^. The poor-to-moderate reliability observed in both systems could have introduced measurement noise that weakened the observed correlation. Our findings should be interpreted with consideration of the measurement resolution and lack of sample heterogeneity rather than as evidence that the tests demonstrate fundamentally different constructs of visual dependence.

We validated our VR‑based RDT against the commonly used PC-based system^9^, where both have used the same established optokinetic stimulation of rotating dots at a fixed speed. There have been other VR systems that explore visual dependence, and their validity has been measured against another test, the bucket test. Neither of these tests included dots, they were simply repositioning of a rod to vertical. Similarly, the correlation between these tests was moderate (*r* = 0.46)^54^, highlighting that delivery of a virtual version of a physical test is moderately correlated.

Other tests for assessing subjective visual vertical have been developed, such as a boat in the sea^55^, or variations on the RDT^56,57^. Although validity has been tested in some studies^54,58^, they have been assessed against the bucket test. This reflects a lack of consensus as to which test should be regarded as the “gold-standard”.

If a test is to be used in patient populations to study visual vertical, it has been shown that a dynamic aspect should be included to provide higher sensitivity in differentiating them from healthy people^59^. Our virtual reality version of the test includes such an element (i.e. moving dots) and therefore may be more useful in clinical settings.

### Reliability

The intra-rater reliability varied across the two systems and three positions: the PC-based repeatability was poor to moderate across the three positions (ICC = 0.26–0.47), whereas the VR-based test was poor (ICC = 0.02–0.16). The level of repeatability may be due to measurement sensitivity, with small, and maybe clinically irrelevant differences, amplifying small variations between repeated measurements and consequently lowering the ICC. It may also be due to the varied level of attention by the individuals across the duration of the recording sessions, which lasted approximately 60 min in total with 8 min per RDT. However, it should be noted that excellent repeatability of a VR-based test has been reported before (ICC = 0.94)^29^ and this is likely due to differences in the way the data were analysed. The earlier work excluded test-retest SVV values exceeding ± 2 standard deviations^29^, whereas we retained the complete dataset, excluding only clear setup errors, such as initial rod angles outside of 20° to 70°.

This study also provides the first evidence of reliability of the PC-based RDT, which is only weak to moderate (ICCs = 0.26, 0.35, 0.47). This may be due to us having increased the level of sensitivity of the PC-based test to match that of the VR-based test (0.1° incremental adjustments for the rod). This likely amplified small variations between repeated measurements and consequently lower the ICCs. Additionally, the ICC is highly sensitive to the homogeneity of the sample; in our study, the narrow range of visual dependence (0.9° to 5.3°) among participants also likely constrained the ICC values. In other words, when subjects perform very similarly, the variance between people is small relative to the “noise,” which reduces the ICC.

### Measurement precision

The coexistence of a low ICC and a small SEM in the PC-based RDT warrants consideration. Despite the low ICC, the small SEM (< 0.5°) and MDC (1.2°−1.3°) indicate that the PC-based system has high absolute precision. Clinically, this means the PC device seems to be stable for tracking a single person over time; any change exceeding 1.3° might be viewed as a clinically relevant shift, even though the tool struggles to “rank” healthy individuals due to their similar visual dependence performance.

Since the reference test (PC) itself showed limited test-retest reliability in this cohort, it may set a “ceiling” for the convergent validity of the VR system. SEMs across positions using VR were ~ 0.7°, suggesting it is currently more susceptible to noise compared to the PC version (SEM < 0.5°). The increased susceptibility of the VR system to measurement noise may be attributed to the physical weight of the head-mounted display, which can impact postural stability and head-positioning consistency^60^. To our knowledge, this is the first study to report the SEM for the PC-based RDT. As noted in recent studies^53–56^, the focus of RDT tool development has largely been on mean differences between groups rather than the specific parameters required to assess individual-level measurement reliability.

The MDC in visual dependence measured using VR was between 1.8° and 2.2°; that is, to be considered real a change of approximately 2° is required. The visual dependence measured using the PC had a smaller MDC of between 1.2° and 1.3°. Whether such small differences have functional consequence is uncertain, however, the difference in visual dependence between healthy people and people with balance problems is greater than our MDC^9^. There is currently no consensus on the Minimal Clinically Important Difference for the RDT in the HSD/hEDS population; however, our findings suggest that these small variations may lack scientific meaning for clinical differentiation at this stage.

### Sensory considerations

To explore the impact of alterations to the vestibular system, visual dependence has previously been measured in neutral and lateral flexion of the head^27,29,30^. However, we are interested in the impact of alterations to proprioception, which is the case for people with hypermobility^61^. Therefore, this study extended tests of visual dependence to include head rotation in the yaw plane. The use of VR is particularly helpful when altering head position, as well as testing in other contexts, as it is not as easy to explore visual dependence when restricted to looking through a cone towards the screen of a PC.

## Limitations

A limitation of this study is the poor-to-moderate reliability of the PC-based RDT, which served as our reference standard. The reliability of a test impacts the degree to which the test can be valid^62^. As the reference test did not have good reliability, this would have introduced statistical noise, weakening the observed convergent validity of the VR-based system. Additionally, the high measurement resolution used (0.1°) and the relatively low levels and narrow range of visual dependence in our sample may limit the generalisability of these results. In populations with more severe sensory integration deficits (e.g. those with vestibular disorders), a broader range of visual dependence might yield different psychometric properties and stronger correlations. The time of testing was not controlled in our study, although there is some evidence that that precision in SVV is less immediately after waking^63^. However, this was not relevant in our study as participants had to travel to our facility for testing. In addition, cognitive load was not measured either subjectively (via questionnaires) or objectively (via reasoning tests), although it may influence engagement with tests involving VR^29^. Regarding our sample size, it should be noted that our a priori sample size calculation for the validity arm of the investigation was 29. It was not calculated taking into account the combined analyses of validity and reliability, even in the context of a reliability study being sufficiently powered with only 15 participants. Finally, our sample was 80% female. While some literature suggests sex differences in visual dependence^64^, this distribution reflects the higher prevalence of HSD/hEDS in females. Future studies should include more balanced cohorts to ensure broader generalisability.

## Conclusion

VR-derived measures of visual dependence using the RDT have weak to moderate convergent validity with the PC-derived measure. There is poor test-retest reliability across the three head positions. Correlations appear constrained by measurement precision and dependent upon recruiting people with a broad range of visual dependences. The differences when the test was repeated led to poor intra-rater reliability, but this may not be functionally significant. Caution should be taken in interpreting reliability using statistical testing without assessing whether these differences are clinically meaningful.

## Supplementary Information

Below is the link to the electronic supplementary material.

Supplementary Material 1

## Data Availability

The datasets used during the current study are available in Additional file 1.

## Abbreviations

VR: Virtual reality
RDT: Rod and disk test
PC: Personal computer
HSD: Hypermobility spectrum disorders
hEDS: hypermobile ehlers danlos syndrome
CI: Confidence interval
ICC: Intraclass correlation coefficients
SD: Standard deviation
SEM: Standard error of measurement
MDC: Minimal detectable change
CSQ-VR: The cybersickness in VR questionnaire
